# Supplementary material for: Bortezomib Eliminates Persistent Chlamydia trachomatis Infection through Rapid and Specific Host Cell Apoptosis
Source: Int J Mol Sci. 2022 Jul 4;23(13):7434. doi: 10.3390/ijms23137434 (PMC9267172; doi:10.3390/ijms23137434)
Supplement: Supplementary file 1 [file ijms-23-07434-s001.zip › ijms-1771329-supplementary.pdf]

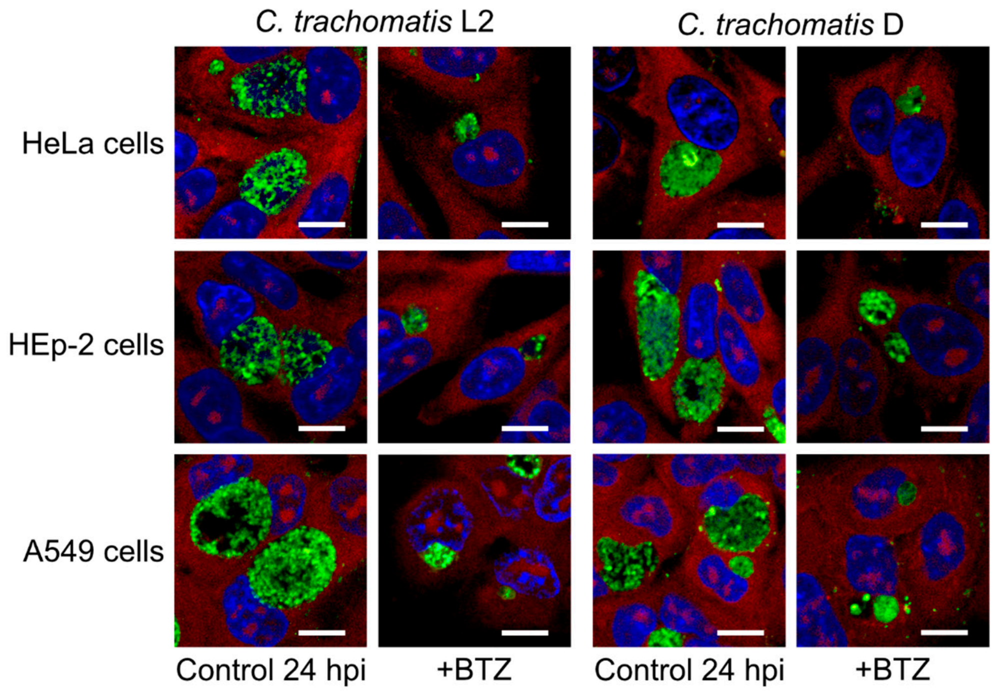

Figure S1. HeLa, HEp-2 or A549 cells were infected with *C. trachomatis* L2 or D respectively, and cultured with or without 1 μM BTZ for 24 h. After fixation, cells were stained using a FITC-conjugated anti-*Chlamydia* LPS antibody. Fluorescence images were acquired using a Zeiss LSM710 confocal microscope with Zeiss ZEN 2010 acquisition software. Red stain denotes Evans blue counterstaining. Blue denote DAPI nuclear staining. Scale bar, 10 μm.

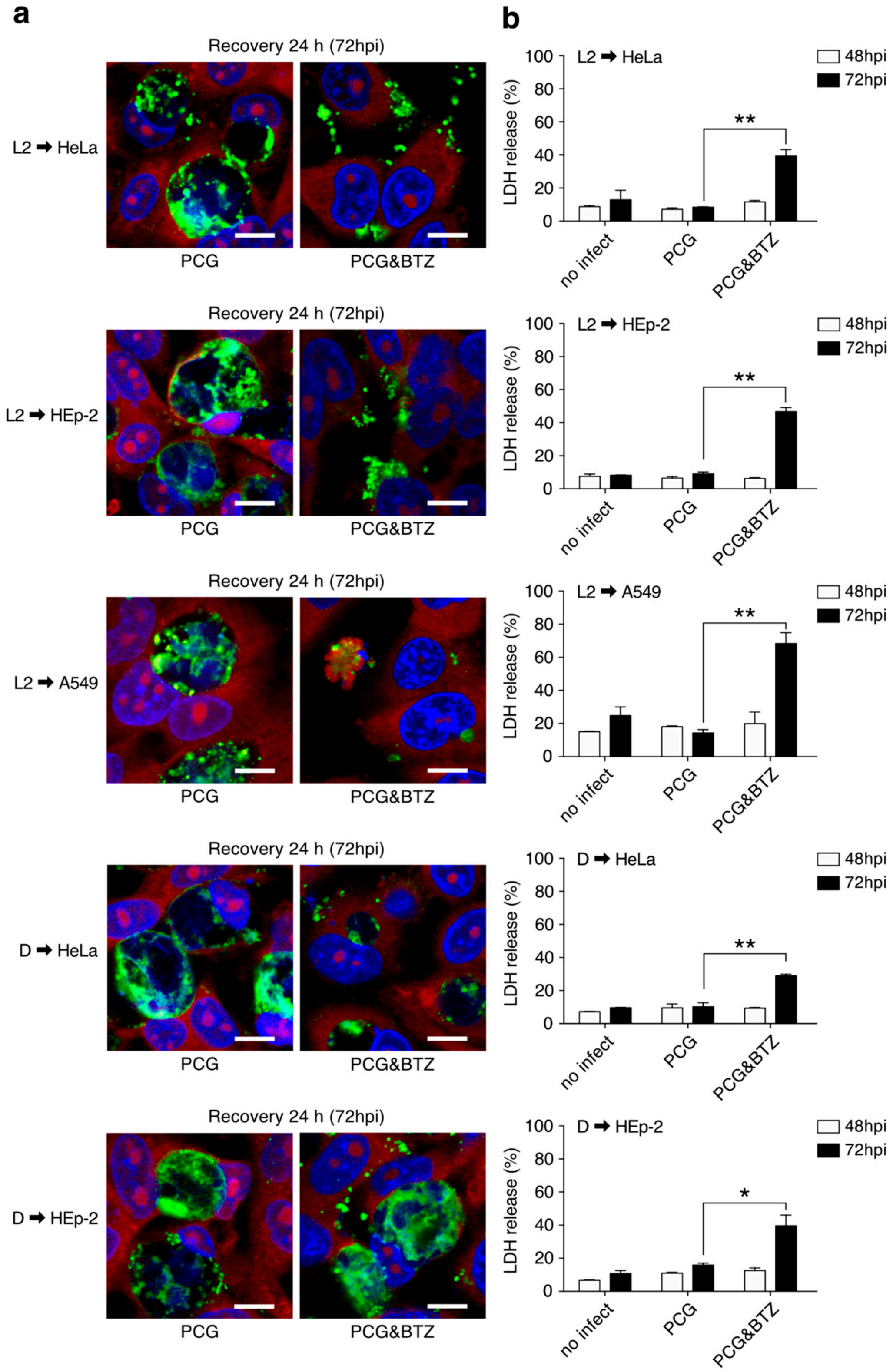

Figure S2. (**a**) HeLa, HEp-2 or A549 cells were infected with *C. trachomatis* serovar L2, or D, respectively, and were treated with 5 units/mL PCG alone, or PCG and 1μM BTZ, as indicated time course in Figure 5a. Cells were fixed at 72 hpi (recovery 24 h), and stained using a FITC-conjugated anti-*Chlamydia* LPS antibody. Fluorescence images were acquired using a Zeiss LSM710 confocal microscope with Zeiss ZEN 2010 acquisition software. Red stain denotes Evans blue counterstaining. Blue denote DAPI. Scale bar, 10 μm. (**b**) Each culture supernatant was collected at indicated time, and the released LDH activity was measured. The total cell lysate of each cell line was used as a 100% cytotoxicity control. Data are the mean ± *SD* of three independent wells. *$p < 0.05$; **$p < 0.01$, by Welch's *t*-tests.
